# Extreme ultraviolet resonant inelastic X-ray scattering (RIXS) at a seeded free-electron laser

**DOI:** 10.1038/srep38796

**Published:** 2016-12-12

**Authors:** M. Dell’Angela, F. Hieke, M. Malvestuto, L. Sturari, S. Bajt, I. V. Kozhevnikov, J. Ratanapreechachai, A. Caretta, B. Casarin, F. Glerean, A. M. Kalashnikova, R. V. Pisarev, Y.-D. Chuang, G. Manzoni, F. Cilento, R. Mincigrucci, A. Simoncig, E. Principi, C. Masciovecchio, L. Raimondi, N. Mahne, C. Svetina, M. Zangrando, R. Passuello, G. Gaio, M. Prica, M. Scarcia, G. Kourousias, R. Borghes, L. Giannessi, W. Wurth, F. Parmigiani

**Affiliations:** 1 Elettra Sincrotrone Trieste S.C.p.A., Strada Statale 14 - km 163.5, 34149 Trieste, Italy; 2 CNR-IOM, Strada Statale 14 - km 163.5, 34149 Trieste, Italy; 3 Physics Department and Center for Free-Electron Laser Science, Hamburg University, 22607 Hamburg, Germany; 4 Photon Science, DESY, Notkestr. 85, 22607 Hamburg, Germany; 5 Shubnikov Institute of Crystallography, Russian Academy of Sciences, Leninsky pr. 59, Moscow 119333, Russia; 6 Cavendish Laboratory, University of Cambridge, JJ Thomson Avenue, Cambridge CB3 0HE, UK; 7 Physics Department, University of Trieste, Via Valerio 2, 34127 Trieste, Italy; 8 Ioffe Physical-Technical Institute, Politekhnicheskaya 26, 194021 St. Petersburg, Russia; 9 Advanced Light Source, Lawrence Berkeley National Laboratory, Berkeley, California 94720, USA; 10 International Faculty, University of Köln, 50937 Köln, Germany

## Abstract

In the past few years, we have been witnessing an increased interest for studying materials properties under non-equilibrium conditions. Several well established spectroscopies for experiments in the energy domain have been successfully adapted to the time domain with sub-picosecond time resolution. Here we show the realization of high resolution resonant inelastic X-ray scattering (RIXS) with a stable ultrashort X-ray source such as an externally seeded free electron laser (FEL). We have designed and constructed a RIXS experimental endstation that allowed us to successfully measure the d-d excitations in KCoF_3_ single crystals at the cobalt M_2,3_-edge at FERMI FEL (Elettra-Sincrotrone Trieste, Italy). The FEL-RIXS spectra show an excellent agreement with the ones obtained from the same samples at the MERIXS endstation of the MERLIN beamline at the Advanced Light Source storage ring (Berkeley, USA). We established experimental protocols for performing time resolved RIXS experiments at a FEL source to avoid X ray-induced sample damage, while retaining comparable acquisition time to the synchrotron based measurements. Finally, we measured and modelled the influence of the FEL mixed electromagnetic modes, also present in externally seeded FELs, and the beam transport with ~120 meV experimental resolution achieved in the presented RIXS setup.

High resolution resonant inelastic X-ray scattering (RIXS) is a powerful X-ray spectroscopy for studying the low-energy charge, spin, orbital and lattice excitations in solids. In the two-step model describing the RIXS process, these elementary excitations are created by the inelastically scattered X-rays from the sample^1^. The system is resonantly excited by X-ray photons from the ground state to a core-excited intermediate state and then relaxes back to a low-energy excited final state. By measuring the energy difference between incoming and outgoing photons from the sample, a detailed map of low energy excitations can be obtained. At present, RIXS measurements are routinely performed at high brilliance synchrotron radiation facilities. The most advanced RIXS spectrometers allow the detection of elementary excitations with an energy resolution of few tens of meVs^2^,^3^,^4^.

With the advent of free electron lasers (FELs) providing high brightness sub-picosecond X-ray pulses, one can envision to employ RIXS in ultrafast time resolved experiments. Traditionally pump-probe optical experiments use an optical pulse to excite the samples (pump) and a time delayed optical pulse (probe) serves as a stroboscopic device to observe the sample relaxation back to equilibrium. In this respect, RIXS can be employed to map out the temporal evolution of low-energy excitations in the sub-picosecond timescale. For this, suitable sub-ps X-ray pulses are required as a probe. Several time-resolved resonant X-ray emission (RXES) or RIXS experiments have been carried out at FEL facilities with very low to moderate energy resolution, making use of only the resonant enhancement and the element specificity in the RIXS process^5^,^6^,^7^. To our knowledge, there is only one publication on FEL-based high resolution RIXS experiment to study the low energy excitations in correlated materials^8^. This experiment by Rusydi *et al*. was performed at the PG1 beamline of FLASH FEL (DESY, Hamburg), with the typical SASE (self amplified spontaneous emission)-FEL pulses that have an energy bandwidth on the order of 10^−1^ (ΔE/E). This bandwidth dramatically limits the overall RIXS energy resolution. In order to achieve high energy resolution for RIXS measurements with SASE FEL, a monochromator like that of PG1 beamline at FLASH is required to narrow the energy bandwidth. However, energy filtering SASE pulses leads to strong shot-to-shot intensity fluctuations (up to 100% rms) where the low intensity X-ray shots do not provide useful information^9^. Hence, extended data acquisition times are needed to achieve a suitable signal statistics to analyze RIXS spectra. To overcome this problem, an externally seeded FEL, like FERMI (Elettra, Trieste), can be used since it generates quasi-transform limited pulses with stable photon energy and extremely narrow bandwidth (on the order of ~10^−3^) at pulse-to-pulse intensity fluctuation below 20% rms^10^. With such stable source, the as generated photons can be used for the experiments, i.e. the monochromator is no longer needed, and the averaged photon flux along with the pulse bandwidth are suitable for time resolved high resolution RIXS experiments.

RIXS is commonly performed in the soft X-ray regime, e.g. across the transition metal L-edges, and there is an undergoing continuous development of beamlines and advanced X-ray spectrometers to achieve high spectral resolution in this energy range^1^,^11^. Several soft X-ray RIXS instruments (or beamlines) under development at the LCLS (Stanford, USA) and European XFEL (Hamburg, Germany) will soon be operational. However, it has been shown recently that the extension of RIXS into the EUV energy range (10 eV < hν < 120 eV) is of great advantage for studying certain classes of materials^12^,^13^,^14^. At these photon energies, high energy resolution can be achieved even with commercial spectrometers. Since this energy range well matches the FERMI FEL-1, the aim of present work is to establish the high resolution EUV RIXS spectroscopy at the FERMI FEL. To attain this goal, we have built a novel experimental endstation that can be used at the TIMEX beamline operated with FERMI FEL.

In the following, we show that the ~1 eV d-d excitations in KCoF_3_ can be measured with RIXS at cobalt M_2,3_-edge. The FEL RIXS data are compared with very high resolution M-edge RIXS spectra measured on the same sample at the Advanced Light Source (ALS), Lawrence Berkeley National Laboratory. The comparison shows an excellent agreement between FEL- and synchrotron-RIXS spectra. We establish the experimental conditions to mitigate the potential sample damage by the intense FEL pulses, while achieving signal statistics comparable to that obtained from the synchrotron experiments. The final energy resolution achieved in our experiments was ~120 meV. Furthermore, we have shown that possible spurious FEL emission modes transported through the beamline can produce unwanted components around the elastic line, which can extend in the present case up to ~500 meV in energy loss. However, by an in-depth analysis of RIXS spectra and EUV ray-tracing, we unraveled the origin of such spectral artifacts and managed to minimize their contribution to the RIXS signal. The results presented here are important steps on the road towards time-resolved EUV RIXS experiments at seeded FELs with high energy and time resolution.

## Results

**Figure 1:**
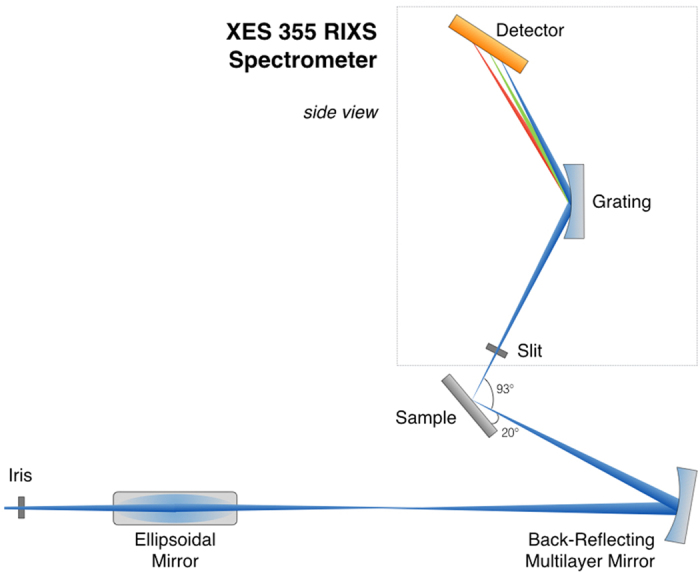
Scheme of the experimental setup. The FEL beam from the fixed focus ellipsoidal mirror is collected and refocused on the sample by a multilayer mirror in back-reflection geometry. The FEL photon pulse polarization is vertical, i.e. in the scattering plane. To simplify the scheme, the XES 355 has been rotated in the figure by 90 degrees around the vertical with respect to the real setup.

The RIXS setup at TIMEX beamline of FERMI FEL is schematically illustrated in Fig. 1. The setup installed after the last ellipsoidal mirror of TIMEX beamline comprises a back-reflecting multilayer mirror for x-ray focusing and a commercially available X-ray emission spectrometer (XES 355). (More details can be found in the Methods section). Resonant elastic scattering (RXES) was used to determine the resonance conditions, i.e. the excitation photon energies for RIXS measurements. RXES is very strong in the EUV regimes and it yields intense and unavoidable off-specular reflectivity that produces an elastic line in the RIXS spectra normally several orders of magnitude stronger than the inelastic features. Such strong elastic line often obscures the signal in the few hundred meV energy loss range^12^.

**Figure 2:**
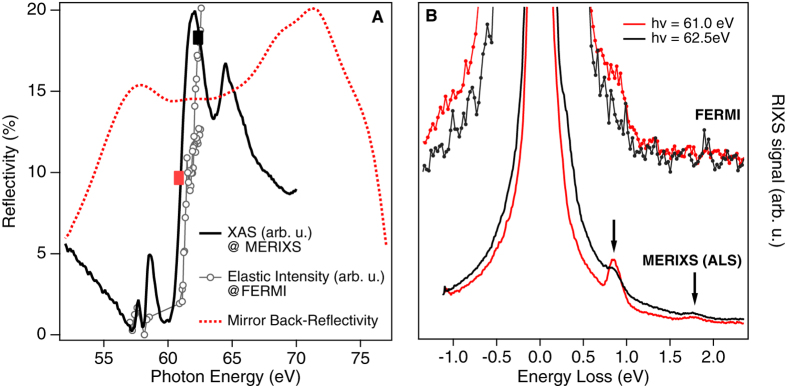
(**A**) Solid Line: Cobalt M_2,3_-edge X-ray absorption (XAS) spectrum of KCoF_3_ measured in TEY mode at MERLIN beamline at the ALS. Line and markers: Intensity of the diffuse elastic line measured at FERMI as a function of incoming photon energy. Each point represents the total number of counts measured by the CCD detector averaged over 1000 FEL shots, while the FEL photon energy was varied via an automatized scanning procedure. The gap between 59 eV and 61 eV in the RXES curve is caused by the switching of seed harmonics where the automatized procedure for FEL tuning has not been optimized to deliver X-rays. The data have been normalized by the incoming photon flux, which is determined by measuring the shot-by-shot current on the last TIMEX beamline mirror (ellipsoidal), taking into account the energy-dependent reflectivity of the back-reflecting mirror included in the RIXS setup. The red and black squares mark the two photon energies at which the RIXS signal has been measured. Dashed red curve: Reflectivity of back-reflecting refocusing multilayer mirror as a function of incoming photon energy. The reflectivity shown is from the center of the mirror but is uniform across the mirror. (**B**) RIXS spectra measured at MERIXS (solid lines) and at FERMI (lines and dots) at two excitation energies 61.0 eV (red) and 62.5 eV (black). The resonating d-d transition (solid arrow) at about 1 eV can be identified in both measurements despite the different energy resolution of these two measurements.

Figure 2A shows the cobalt M_2,3_-edge XAS spectrum from KCoF_3_, as measured at the ALS (solid line), and the RXES measured at FERMI FEL (markers). Both measurements clearly show the onset of cobalt M_2,3_-edge with the RXES signal shifted to higher photon energies with respect to the XAS. As in the case of NiO, this shift can be attributed to the strong Fano interference between resonant and non-resonant scattering channels, generating a dip at the leading edge of the resonant intensity and causing the energy shift in the elastic features^15^,^16^. Once the cobalt M_2,3_-edge has been identified, a prolonged data acquisition was performed to reveal the inelastic features (EUV RIXS spectra) at two resonant photon energies: 61 eV (red square, Fig. 2A) and 62.5 eV (black square, Fig. 2A). The FERMI RIXS spectra, shown in Fig. 2B as dotted lines, are compared with the high resolution RIXS spectra from MERIXS (solid lines). The d-d excitation at ~1 eV energy loss can be clearly seen in both data sets. In particular, the RIXS spectra from MERIXS resemble those measured on CoO with inelastic features at ~1 eV and ~1.8 eV (solid arrows in Fig. 2B)^17^,^18^. The inelastic features at ~1.8 eV energy loss are below the background in the FERMI data. The FERMI spectra at 61 eV and 62.5 eV were obtained by integrating over 123600 and 36000 single shots, corresponding to 206 and 60 minutes acquisition time respectively in the 10 Hz operation mode of FERMI. The acquisition time for both sets of spectra at MERIXS was 90 minutes. The acquisition time at FERMI will be strongly reduced in the recently introduced 50 Hz operation mode. It can be further reduced with a dedicated X-ray spectrometer with larger acceptance angle designed for the specific FEL source characteristics.

**Figure 3:**
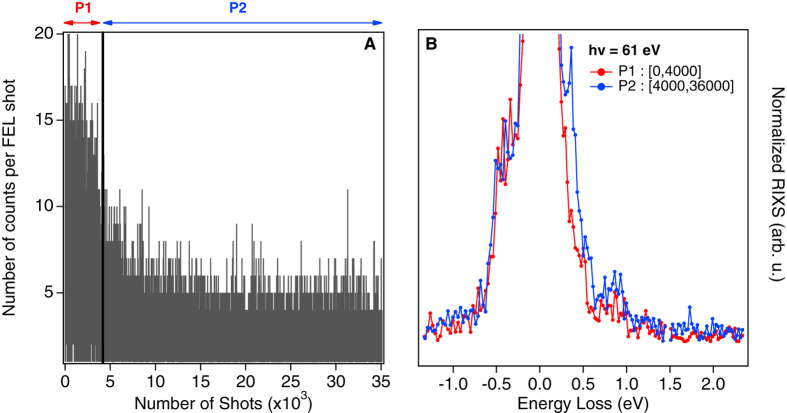
(**A**) Number of counts detected per FEL shot, proportional to the RXES signal, as a function of number of shots with 6 μJ/pulse FEL. After the first few thousand shots, the count rate decreases indicating the sample damage. The RIXS signal at 1 eV (**B**) is still present when summing the first 4000 and the last 32000 measurements. The elastic lines in the spectra are normalized to unity.

The spectra in Fig. 2B were obtained by averaging the FEL shots with pulse energies up to 5 μJ/pulse. After taking into account the overall beamline transmission and the reflectivity of back-reflecting mirror shown in Fig. 2A, the average fluence on the sample was estimated to be 0.7 J/cm^2^. This value can be increased to reduce the acquisition time. However for pulse energies higher than 6 μJ/pulse, the KCoF_3_ sample undergoes an irreversible structural change. Figure 3A shows that the number of detected counts per FEL shot proportional to the RXES signal at an average pulse energy of 6 μJ varies with the number of shots on the sample. We attribute this effect to radiation “damages”. Independent of the RXES intensity drop, the ~1 eV d-d excitations remain visible in the RIXS spectra obtained by summing the first 4000 shots (P1) and the last 32000 shots (P2) (Fig. 3B). However additional features appear on the energy loss side of elastic emission and the RIXS features are submerged in this background. For all further analysis, we considered only datasets where the total number of counts per pulse was not changing.

## Discussion

**Figure 4:**
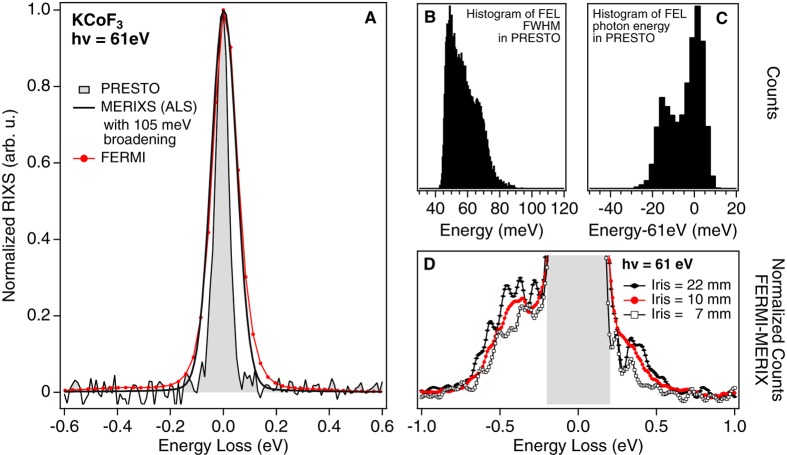
(**A**) Elastic line measured at FERMI (red line) and at MERIXS (solid black line with additional 105 meV Gaussian broadening) on KCoF_3_. The resolution of the FERMI setup was 120 meV, evaluated from the FWHM of the elastic line. The filled trace represents the energy distribution of FEL integrated over the full measurement obtained by means of the on-line spectrometer PRESTO for FEL diagnostics. This comparison shows that the resolution is limited by the spectrometer settings and not by the FEL energy distribution. (**B** and **C**) Histograms of the FWHM and relative shift of the FEL photon energy determined for each shot in the measurement. (**D**) Elastic line tails in the FERMI data for different settings of the diameter of the beamline iris aperture. The datapoints show the difference between the normalized FERMI RIXS spectrum and the broadened MERIXS spectrum in Fig. 4A.

We have demonstrated that high-resolution EUV RIXS experiments are feasible at seeded FELs. This challenging experiment was made possible only with the highly stable and quasi-transform limited light pulses from externally seeded FELs. Figure 4A shows that the energy resolution evaluated from the full width of the elastic line in the FERMI RIXS spectra is ~120 meV. Compared with the high resolution measurements performed at MERIXS, there is an additional 105 meV resolution broadening (Fig. 4A).

The commercial spectrometer (XES 355) employed at FERMI presents one order of magnitude worse resolution than the MERIXS custom spectrometer we used at ALS. Taking into account the spectrometer settings for the measurements, we actually expected an energy resolution of ~170 meV. We suppose the improved resolution observed at FERMI depends on the fact that the FEL spot on the sample dispersive direction was significantly smaller than the spectrometer entrance slit (~50 μm)^19^. Contributions to the energy resolution broadening are due to the FEL pulse energy bandwidth, FEL beam mode mixing, and the XES 355 spectrometer resolution associated to the reduced FEL beam size with respect to the slit aperture. To study the FEL bandwidth contributions, we considered the shot-by-shot energy distribution in the FEL measured by the on-line spectrometer PRESTO installed in front of the TIMEX beamline^20^. We carried out the shot-by-shot analysis of 36000 FEL shots recorded during the RIXS measurements. Figure 4A shows the average energy distribution of these 36000 FEL shots. Interestingly, the FWHM of FEL pulses before the photon beam transport, is ~60 meV. This value is consistent with quasi-transform limited pulses and is found to be significantly smaller than the overall ~120 meV experimental resolution in the RIXS spectra. Not all 36000 FEL shots are equivalent. Figure 4B and C show the histograms of FWHM and energy position obtained using the Gaussian fitting on the elastic line in the shot-by-shot analysis. The maximum broadening of the elastic line expected from the FEL bandwidth is less than ~80 meV, while the photon energy shift is less than ~20 meV. Therefore we conclude that the major contribution to the overall experimental resolution is from the RIXS spectrometer.

Figure 4D shows the tails of the elastic line obtained by subtracting the MERIXS RIXS spectra (solid black line in Fig. 4A) from the FEL RIXS spectra (red line and markers in Fig. 4A). For this subtraction, a 105 meV broadening was applied to the elastic line of MERIXS RIXS spectra to attain a comparable resolution between two experiments. The difference signal highlights several side features around the elastic line and their spread to ~500 meV energy loss. Interestingly, the intensity and shape of these features depend on the settings of the iris aperture in the beam path (Fig. 1). This suggests that the measured FEL RIXS spectra are also affected by artifacts from unwanted optical effects in the photon beam transport and/or from the transversal spatial intensity distribution of FEL pulses. To clarify this statement, it is important to note that all FEL optical devices (beamline and endstation) are based on the assumption of Gaussian optics, i.e. TEM_00_ mode. Any deviation from this assumption will influence the photon beam intensity distribution on the sample and finally the RIXS spectra and the achievable RIXS resolution.

**Figure 5:**
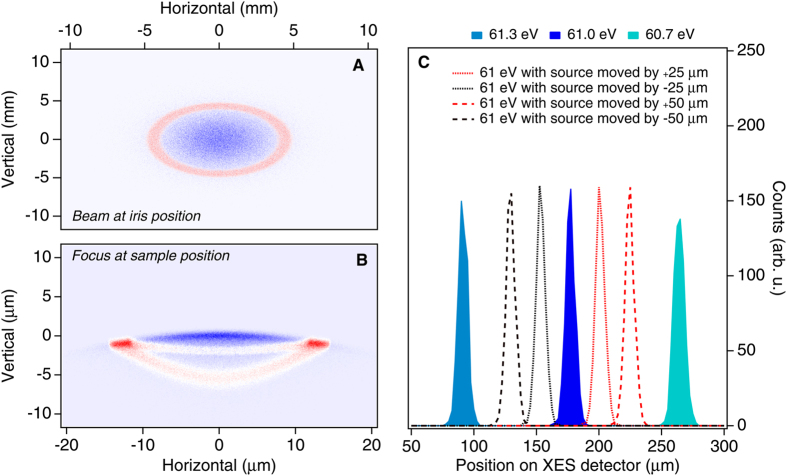
(**A** and **B**) X-ray spot at the iris position (**A**) and at the sample position (**B**) obtained by a ray-tracing simulation with SHADOW. We considered a 61 eV X-ray beam with a spatial distribution comprising an inner core (blue area) and an annular mode (red area). (**C**) Ray-tracing of 61.0, 62.3 and 60.7 eV beams in the XES 355 spectrometer from the ideal focus of the 300 lines/mm grating (filled curved) and from a source point displaced laterally with respect to the ideal focus by 25 and 50 μm in both directions.

We performed a ray-tracing simulation using the SHADOW code^21^,^22^. In the simulation, we included the TIMEX ellipsoidal mirror and the back-reflecting mirror of the RIXS endstation (see Fig. 1). We considered a mixed-modes operating condition with a light spot intensity distribution at the iris position as simulated in Fig. 5A, where the Gaussian mode is shown in blue and a hollow mode as shown in red. The hollow mode may be generated by diffraction along the beam transport or by the FEL itself^20^. The FEL may indeed generate mixed transverse modes characterized by the presence of a superimposed hollow mode. This is originated from the off-axis resonant condition of the last FERMI undulators, when detuned, e.g. for efficiency enhancement. The chosen distribution is a simplification of the real X-ray beam profile, to reproduce intensity inhomogeneities that are crucial in the analysis. Figure 5B shows the focus at the sample location projected in the vertical plane. The focal size in the dispersive direction is assumed to be about 20 μm, i.e. significantly smaller than the spectrometer slit aperture settings. However, propagating the FEL beam onto the sample, the annular mode component produces a structure with side maxima, as shown by the red spots in Fig. 5B. Finally, Fig. 5C reports the simulated effects of mixed-mode operating conditions on the RIXS spectrum. In the simulation, the X-ray beams have been propagated into the RIXS spectrometer for three photon energies, 61.3, 61.0 and 60.7 eV using an ideal Gaussian beam. The difference in the FEL photon energy gives rise to different positions for the elastically scattered beam on the detector. To simulate the hollow mode, i.e. the red spots in Fig. 5A, the 61 eV beam has been moved from the central position at the slit coordinate by ±25 and ±50 μm. This leads to a significant displacement for the measured features on the XES detector as shown in Fig. 5C by the dashed lines. These additional structures can be responsible for the spurious elastic side contributions around the main Gaussian elastic emission. Their relative intensity with respect to the main line is determined by the relative intensities of different FEL modes and is not considered in the simulation. The RIXS spectra in Fig. 4D acquired with three different iris apertures of 22, 10 and 7 mm diameter substantiate the simulation: the intensity and shape of the features around the elastic emission depend on the iris aperture settings. This indicates how the measured RIXS FEL spectra are affected by the transversal intensity distribution of the pulses at the spectrometer entrance slit.

Notwithstanding the high energy resolution of the FEL pulses, the beam focal properties at the slit or intensity inhomogeneities due to a mode composition that deviate significantly from the Gaussian profile of the X-ray spot, can significantly affect the RIXS spectra. A good control over the FEL operating mode and transport is essential to maximize the RIXS spectra resolution and can be achieved in an iterative way by using RIXS measurements as a guide during the machine tuning.

## Conclusions

We have measured for the first time the high resolution EUV RIXS at a seeded FEL on a KCoF_3_ single crystal. To achieve this goal, we developed an experimental endstation comprising a back-reflecting mirror and a commercial XES 355 spectrometer used the TIMEX beamline of FERMI FEL. We were able to measure the d-d excitations from KCoF_3_ using EUV RIXS spectroscopy with ~120 meV resolution in an overall detection time comparable to the synchrotron measurements. These results have been made possible with the near-transform limited seeded FEL pulses that provide a very stable radiation source as compared to the SASE FELs, where the mandatory use of a monochromator for high energy resolution severely limits the throughput and introduces intensity fluctuations. The intensity fluctuation during the RIXS measurement was ~15% rms. In spite of the great advantages offered by the externally seeded FELs for non-linear spectroscopy experiments in the EUV and X-ray spectral regime, the behaviors of FEL electromagnetic modes, the setup of the instrument and the transport of the light to the experiment itself must be carefully considered to fully exploit the energy resolution allowed by the FERMI seeded FEL. The present experiment and data analysis have shown that the side peaks observed around the elastic emission in the FEL-RIXS spectra may be justified by a non-Gaussian mode distribution at the entrance of the instrument, which inevitably mask the genuine RIXS features within several hundred of meV around the elastic emission. This suggests, for high energy resolution measurements (<100 meV for FEL-RIXS experiments), a careful analysis of the beam mode composition and stability at the entrance of the instrument to extract the spectral signal from the spurious background.

## Methods

The sample was a 2000 μm thick single-crystal KCoF_3_^23^,^24^. KCoF_3_ has cubic perovskite structure where Co ions have a nominally 2+ oxidation state. KCoF_3_ is an antiferromagnet with the Neél temperature around 114 K. All measurements presented here were performed in the paramagnetic phase at room temperature. In this phase, the cobalt M_2,3_-edge RIXS spectra of KCoF_3_ resemble the ones from CoO^17^,^18^. Unlike CoO, KCoF_3_ sample is robust and does not require special surface preparation in UHV. In addition, the single crystal is more resilient to higher X-ray fluencies compared to CoO thin films^18^.

Co M_2,3_-edge RIXS measurements were performed at FERMI free-electron laser facility (Elettra, Italy) using the novel RIXS endstation. FERMI FEL 1 delivers vertically polarized X-rays at 20.3 nm (61 eV) and 19.84 nm (62.5 eV) wavelength. To explore the sample damage induced by high X-ray fluence pulses, the photon flux measured by the flux monitors at the entrance of beamline was varied up to 8 μJ per pulse using the combination of beamline Al filters and FERMI N_2_ gas attenuator. X-ray shots with energy up to 5 μJ per pulse did not introduce damage to the sample. The experimental setup is schematically illustrated in Fig. 1. The RIXS endstation was installed downstream from the TIMEX endstation. The X-ray beam, tightly focused at the TIMEX endstation (5 × 5 μm^2^) by the upstream ellipsoidal mirror, was refocused at the sample position by a broad-band multilayer mirror (20 cm focal length) operated at 3° incident angle in the back-reflecting configuration (Fig. 1).

The back-reflecting multilayer mirror was designed and coated at the X-ray multilayer laboratory at DESY (Hamburg, Germany) using magnetron sputtering technique. The multilayer design called for a large energy bandpass covering the range from 56 to 77 eV (16.1–22.1 nm) to be able to simultaneously reflect photons with energies across the M_2,3_-edges of Fe, Co and Ni. The multilayer consisted of 40 Mo and Si layers. A standard periodic multilayer would only have a bandpass of a few eV. To obtain larger bandpass we used an aperiodic multilayer design^25^,^26^ based on the algorithm published in ref. 27. This multilayer was deposited on a 25.4 mm diameter silicon substrate with a radius of curvature of 40 cm prepared by Pilz-Optics. The high spatial frequency surface roughness of the substrate was 0.4 nm rms as measured with atomic force microscope. The final multilayer-coated mirror was measured at-wavelength in the final geometry (3° incident angle) at the Physikalisch Technische Bundesanstalt (PTB) reflectometry beamline at Bessy II in Berlin, Germany. Figure 2A is showing measured reflectivity curve of the mirror used in this experiment between 52 to 77 eV. The X-ray beam size on the sample was estimated to be 20 (H) × 5 (V) μm^2^ using SHADOW ray-tracing simulation. The vertical beam size was larger when projected on the sample at 20° grazing incidence. In order to better control the transverse shape of the beam throughout the beamline (see later discussion), an iris installed before the TIMEX ellipsoidal mirror was inserted during the measurements.

The X-rays scattered from the sample were energy dispersed and recorded using the XES 355 spectrometer (VG Scienta) mounted in the vertical geometry (π-polarization). The 300 lines/mm spectrometer grating operated at the 1^st^ diffraction order was chosen for analyzing the low energy X-ray inelastic emission^19^. The spectrometer detector unit consists of an MCP stack, a phosphor screen, and a CCD. The detector unit has single photon sensitivity and multi-hit capability. The countrate at the used attenuation settings for the X-rays, was in the range of 1–20 counts per pulse. The entrance slit width of the spectrometer was set to 50 μm. With this slit setting, the theoretically determined spectrometer energy resolution is 170 meV. Note, that the slit width is larger than the estimated X-ray spot size on the sample, which increases the experimentally obtained resolution to 120 meV.

Reference XAS and RIXS measurements were carried out at beamline 4.0.3 (MERLIN) RIXS endstation (MERIXS) at the Advanced Light Source (ALS), Lawrence Berkeley National Laboratory. The energy resolution, determined from the FWHM of elastic peak in RIXS spectra was 30 meV. RIXS data were recorded using the slitless VLS based X-ray emission spectrometer equipped with a commercially available in vacuum CCD detector^13^,^14^. The experimental geometry was the same as that of the FEL measurements where the sample was placed at 20° grazing incidence angle relative to the incoming X-ray beam and the photon polarization was maintained in the scattering plane (Fig. 1). XAS measurements were recorded in the total fluorescent yield mode (TEY).

## Additional Information

**How to cite this article**: Dell’Angela, M. *et al*. Extreme ultraviolet resonant inelastic X-ray scattering (RIXS) at a seeded free-electron laser. *Sci. Rep.* **6**, 38796; doi: 10.1038/srep38796 (2016).

**Publisher's note:** Springer Nature remains neutral with regard to jurisdictional claims in published maps and institutional affiliations.
